# Incidence of deep vein thrombosis before and after total knee arthroplasty without pharmacologic prophylaxis: a 128-row multidetector CT indirect venography study

**DOI:** 10.1186/s12891-018-2166-8

**Published:** 2018-07-31

**Authors:** Moon Jong Chang, Min Kyu Song, Min Gyu Kyung, Jae Hoon Shin, Chong Bum Chang, Seung-Baik Kang

**Affiliations:** Department of Orthopedic Surgery, Seoul National University College of Medicine, SMG-SNU Boramae Medical Center, 5 Gil 20, Boramae-road, Dongjak-gu, Seoul, 07061 South Korea

**Keywords:** Venous thromboembolism, Deep vein thrombosis, Total knee arthroplasty, Mechanical compression device, Indirect venography

## Abstract

**Background:**

We sought to document the incidences of deep vein thrombosis (DVT) before and after total knee arthroplasty (TKA). In addition, we aimed to explor whether routine preoperative DVT evaluation was useful to establish DVT treatment strategies after TKA. Finally, we wanted to evaluate whether the incidences of DVT differed between patients undergoing unilateral and staged bilateral TKA within the same hospitalization period.

**Methods:**

The retrospective study included 153 consecutive patients (253 knees) with osteoarthritis who underwent primary TKA. After surgery, mechanical compression devices (only) were used for DVT prophylaxis. DVT status before and after TKA was determined via 128-row, multidetector, computed tomography/indirect venography.

**Results:**

Overall, the preoperative DVT incidence was 2.6% per patient and 1.6% per knee. All preoperative DVTs were distal in nature and asymptomatic. After TKA, newly developed thrombi were evident in various calf veins, without propagation of any pre-existing thrombi. Postoperatively, the overall incidences of DVT were 69.9% per patient and 58.5% per knee. The DVT incidences were 66% per patient and 69.8% per knee in the unilateral TKA group. In contrast, the incidences were 72% per patient and 55.5% per knee in the staged bilateral TKA group. There was one case of symptomatic distal (unilateral TKA; 0.65% per patient and 0.4% per knee) and proximal DVT (bilateral TKA; 0.65% per patient and 0.4% per knee), respectively.

**Conclusions:**

The incidence of symptomatic DVT was low in Asian patients treated with mechanical compression devices alone, although substantial portion of patients had DVT after surgery. Routine preoperative DVT evaluation is probably not necessary; preoperative DVT was rare and of limited clinical relevance. Furthermore, staged bilateral TKA during a single period of hospitalization does not increase the incidence of DVT.

## Background

Deep vein thrombosis (DVT) is a frequent and significant complication after total knee arthroplasty (TKA) [1, 2]. However, the incidence, diagnosis, prevention, and treatment of DVT remain controversial [1, 3–9]. Differences in the DVT incidence between Asian and Western populations may have aggravated the controversies [10–14]. Most recent studies have recorded lower incidences of DVT in Asian patients; routine pharmacological prophylaxis is not recommended [1, 2, 6]. Indeed, studies on the use of mechanical compression devices (alone) to prevent DVT have become more common [1, 2, 6, 15]. However, theoretically, the use of such devices alone, thus without pharmacological treatment, would be less effective than the combination.

It was important to calculate the incidences of DVT before and after surgery in patients undergoing TKA to establish appropriate treatment strategies. A previous study found preoperative, asymptomatic vascular disease in 4.6% of patients scheduled for TKA [16]. Furthermore, 8% of patients had DVT before TKA [17]. Therefore, the incidence of preoperative DVT must be calculated to allow accurate estimation of the incidence after surgery. In addition, a prior history of venous thromboembolism (VTE) is a well-known risk factor for DVT development after surgery [18]. Furthermore, if a preexisting DVT can propagate after surgery, additional prophylactic treatment may be required.

The incidence and characteristics of DVT may differ between patients undergoing unilateral and staged bilateral TKA. Patients who undergo the bilateral procedure during a single hospitalization period may have to endure prolonged immobilization and a longer hospital stay than the former patients. Furthermore, in contrast to those who undergo bilateral TKA in a single operation, patients undergoing the staged procedure are subjected to repeat surgery with additional anesthesia. Several studies have compared the incidences of DVT between patients undergoing unilateral and simultaneous bilateral TKA [19, 20]. However, it is not clear whether staged bilateral TKA during a single period of hospitalization increases the incidence of DVT compared to that after unilateral TKA.

Therefore, we documented the incidence of DVT before and after TKA. In addition, we explored whether routine preoperative DVT evaluation was useful when establishing treatment strategies for DVT developing after TKA. Finally, we examined whether the incidence of DVT differed between patients undergoing unilateral or staged bilateral TKA during a single hospitalization period. We hypothesized that a considerable proportion of patients undergoing TKA would exhibit DVT both before and after surgery, and that routine preoperative DVT evaluation would thus be valuable. We also hypothesized that patients undergoing staged bilateral TKA would develop postoperative DVT more often than patients undergoing unilateral TKA.

## Methods

This retrospective study included 153 consecutive patients (253 knees) with osteoarthritis who underwent primary TKA. From April 2016 to November 2016, 198 patients underwent primary TKA, due to osteoarthritis, at our institution. Of these, 45 were excluded because of previous surgery on the same knee joint (8); the use of anticoagulants (including aspirin) for thromboprophylaxis due to the presence of cardiovascular and/or cerebrovascular disease (30); contraindications for CT angiographic evaluation (2); and refusal to participate in the study (5). Ultimately, 153 patients (253 knees) were included. There were 130 females (85%) and 23 (15%) males of mean age 71.4 years (standard deviation [SD], 6.4 years; range, 53–84 years). Fifty-three patients (34.6%) underwent unilateral TKA and 100 (65.4%) staged bilateral TKA. The mean preoperative height and weight were 153.5 cm (SD, 7.4; range; 132.3–177 cm) and 63.9 kg (SD, 11.0; range, 43–95.2 kg). The mean body mass index was 27.1 kg/m^2^ (SD, 3.8; range, 18.6–44.8 kg/m^2^). No demographic difference including number of comorbidities was apparent between patients undergoing unilateral and staged bilateral TKA (Table 1). The study protocol was approved by our institutional review board.

**Table 1:** Demographic data on patients in the unilateral and staged bilateral TKA groups

Variable	Unilateral TKA group (*n* = 53)	Bilateral TKA group (*n* = 100)	*P* value
Age (years)	71.3(range, 55–84; SD, 6.3)	71.5 (range, 53–84; SD, 6.4)	0.891
Proportion of female patients	45 (84.9)^*^	85 (85)^*^	0.988
BMI (kg/m^2^)	26.9 (range, 20.9–44.8; SD, 4.1)	27.2 (range, 18.6–36; SD, 3.7)	0.684
Proportion undergoing general anesthesia	45 (84.9)^*^	95 (95)^*^	0.063
Operation time (min)	95.4 (range, 66–175; SD, 19)	91.2 (range, 43–140; SD, 16.2)	0.159
Length of hospital stay (days)	13.9 (range, 8–23; SD 2.7)	20.7 (range, 11–32; SD 3.9)	< 0.001
Number of comorbidities^a^	1.4 (range, 0–5; SD, 1.0)	1.3 (range, 0–4; SD, 1.1)	0.331

Data are presented as means or numbers of patients. Ranges, standard deviations, and proportions^*^ are shown in parentheses. *Abbreviations* = *TKA* Total knee arthroplasty, *BMI* Body mass index, *SD* standard deviation

^a^Comorbidities include previous venous thromboembolism, varicose vein and previous operation on the leg, which are the major risk factors for deep vein thrombosis

All surgeries were performed by a single surgeon using a standard medial parapatellar approach with inflation of a pneumatic tourniquet, and all patients underwent identical postoperative rehabilitation. The tourniquet pressure was set to 300 mmHg and the tourniquet was inflated until skin closure was attained. Intramedullary guides were used during surgery on both the femora and tibiae. All TKAs featured placement of a fixed-bearing posterior stabilized prosthesis. All components were fixed with cement. A closed suction drain was inserted into the subcutaneous space, and tranexamic acid was not used. Periarticular injections and intravenous patient-controlled analgesia (PCA) were used to control postoperative pain. Active ankle flexion and extension exercises, and quadriceps strengthening exercises, were encouraged immediately after surgery. On the second postoperative day, after removal of the drain, all patients began walking with a walker, and commenced both active and passive range-of-motion (ROM) exercises. The second operation for those undergoing staged bilateral TKA was performed 1 week later, during the same hospitalization period.

Mechanical compression devices were applied to both lower legs of all patients after surgery. The Kendall SCD™ 700 Sequential Compression System (Kendall, Mansfield, MA, USA) was used, commencing on the day of surgery and continuing for 2 weeks thereafter. The device was discontinued at the time of discharge. For patients who underwent staged bilateral TKA, the device was placed on the day of the first operation, and continued to be used for 2 weeks after the second operation. The device has a sleeve for the leg, surrounded by three air chambers, and covers the lower limb from the ankle to the thigh. The device allows bilateral, sequential, circumferential, and gradient leg compression. All patients were fully compliant with treatment delivered by the device during their hospitalization periods.

We screened for DVT in both legs before and after TKA using 128-row, multidetector computed tomography-indirect venography (MDCT-indirect venography) (Philips Healthcare, Cleveland, OH, USA). The preoperative evaluation was performed in the outpatient clinic within 2 weeks prior to surgery, and the postoperative evaluation was performed on day 4 after surgery for patients undergoing unilateral TKA and on day 4 after the second operation for patients undergoing staged bilateral TKA [5]. All scans were obtained with the patient supine after injection of 2 mL/kg of the Optiray® 350 contrast agent (741 mg/mL) at 4–5 mL/s. Scanning was performed in a craniocaudal direction, from the diaphragm to the foot, 3 min after contrast injection. The slice thickness was 2 mm, the window level 50, and the window width 350.

**Fig. 1:**
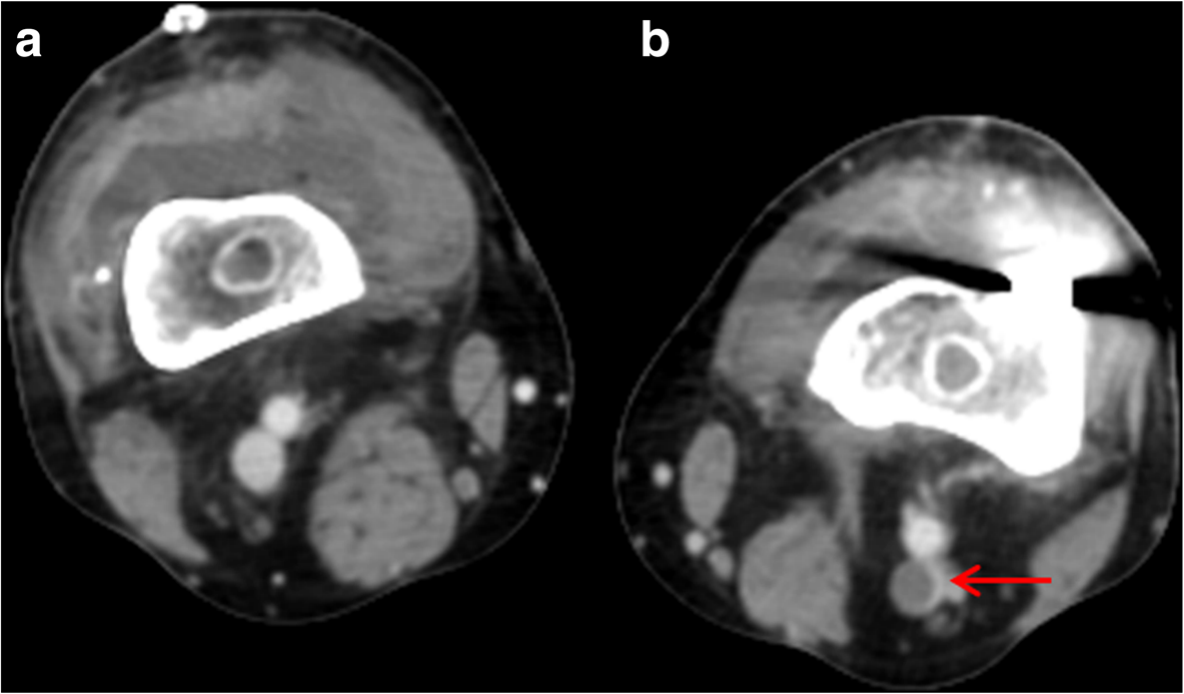
Deep vein thrombosis (DVT) in the left popliteal vein. The image shows that the DVT exhibited a filling defect in the lumen of the vein, surrounded by a highly attenuating enhanced ring

A single radiologist blinded to the study protocol interpreted all venographs. The interpreter was a professor and a board-certified radiologist who had undergone fellowship training. Radiographic evaluation was performed with the aid of a picture archiving and communication system (PACS) (Maroview™, Marotech, Seoul, Korea). On MDCT-indirect venography, a DVT was defined as a low-attenuating partial or complete filling defect in the lumen of a vein, surrounded by a highly attenuating enhanced ring, evident on at least two consecutive images (Fig. 1) [18, 21]. A proximal DVT was defined as a DVT at the level of the popliteal vein or proximal to that vein, and a distal DVT as a DVT involving a calf vein. In principle, we intended to delay surgery if a symptomatic VTE was detected during preoperative evaluation. During the study period, we did not prescribe pharmacological treatment even if an asymptomatic preoperative DVT was evident, based on the recommendation of a previous study on Asian patients [20]. However, if DVT was evident on routine MDCT-indirect venography performed after surgery, we prescribed an anticoagulant regardless of symptoms.

Clinical symptoms and signs of DVT or a PE were recorded by the orthopedic residents in charge. Symptomatic DVT was considered present when DVT was apparent on MDCT-indirect venography, and the patient fulfilled at least one of the following criteria (suggested in a previous study): 1) swelling of all lower extremities; 2) abnormal lower leg swelling; 3) pitting edema; or, 4) a positive Homans’ sign [6]. Imaging to detect PE was performed as described in a previous study; the relevant symptoms were: 1) tachycardia (heart rate > 100 beats/min); 2) dyspnea or tachypnea; 3) sudden chest pain; or, 4) decreased oxygen saturation [6]. Routine clinical follow-up was performed at six weeks, three months, six months, and annually thereafter.

All statistical analyses were performed using SPSS for Windows (version 18.0; SPSS Inc., Chicago, IL, USA) and a *p*-value < 0.05 was considered statistically significant. Demographic data were summarized and compared between the unilateral and staged bilateral TKA groups. Statistical significance was explored using the t-test for two independent samples, and the chi-squared test (or Fisher’s exact test if necessary). The incidences of DVT before and after TKA were described as numbers with percentages. The statistical significance of any difference in the incidence or location (proximal or distal) of DVT between the unilateral and staged bilateral TKA groups was explored using the chi-squared test (or Fisher’s exact test if necessary). The tests obtained more than 80% power within the proper sample size and significance level, α value, of 0.05.

## Results

The overall preoperative incidences of DVT were 2.6% (four patients) per patient and 1.6% (four knees) per knee. All cases of preoperative DVT were distal and asymptomatic. Only one patient had concomitant varicose veins in the index limb prior to surgery. After TKA, they developed new thrombi in different calf veins (three of them also developed new thrombi in the opposite leg) without any propagation to proximal DVT of the preexisting thrombi (Table 2).

**Table 2:** Details of the four patients with preoperative DVT

Case	Index knee	Preoperative DVT	Postoperative DVT
Case 1	Right	Right calf muscle vein	Right peroneal and calf muscle veins
Case 2	Left	Right calf muscle vein	Left below knee veins
Case 3	Right	Left calf muscle vein	Right peroneal, posterior tibial and calf muscle vein, Left calf muscle vein
Case 4	Right	Left calf muscle vein	Right tibioperoneal and calf muscle vein

*Abbreviations* = *DVT* Deep vein thrombosis

Postoperatively, the overall incidences of DVT were 69.9% (107 patients) per patient and 58.5% (148 knees) per knee, respectively. There was one case of symptomatic distal (unilateral TKA; 0.65% per patient and 0.4% per knee) and proximal DVT (bilateral TKA; 0.65% per patient and 0.4% per knee), respectively. The incidences of proximal DVT were 9.8% (fifteen patients) per patient and 7.1% (eighteen knees) per knee, respectively. Only one symptomatic PE with proximal DVT in a unilateral TKA was noted.

The incidence of DVT after TKA did not increase in the staged bilateral TKA group than the unilateral TKA group. The incidences of DVT were 66% (35 of 53) per patient and 69.8% (37 of 53) per knee in the unilateral TKA group. In contrast, the incidences were 72% (72 of 100) per patient and 55.5% (111 of 200) per knee in the staged bilateral TKA group (per patient, *p* = 0.444; per knee, *p* = 0.021). In the unilateral TKA group, 88.6% (31/35) of DVT cases were diagnosed in the operated limb, and 5.7% of DVT cases were (2/35) bilateral DVT. In contrast, bilateral DVT was evident in 54.2% of patients (39/72) in the staged bilateral TKA group. In the bilateral TKA group, the incidence of DVT did not differ between the first and second TKA (55% vs. 56%; *p* = 0.887). In contrast, patients with DVT in the first-operated limb developed DVT more frequently in the second-operated limb (37 of 54 patients) compared with those with no DVT in the first-operated limb (17 of 46 patients) (*p* = 0.001).

## Discussion

The guidelines of the American Academy of Orthopedic Surgeons (AAOS) state that “Patients undergoing elective hip and knee arthroplasty are already at high risk for venous thromboembolism.” [22] Nonetheless, no consensus exists in terms of any of the incidence, diagnosis, prevention, or treatment of DVT, especially in Asian patients [10, 11, 23]. In the present study, we sought to accurately determine the incidences of DVT pre- and post-TKA using 128-row MDCT-indirect venography. In addition, we explored whether routine preoperative DVT evaluation was useful when planning postoperative treatment strategies. Finally, we examined whether the incidence and characteristics of DVT differed between those undergoing unilateral and staged bilateral TKA. Our principal findings were: 1) the incidence of preoperative DVT was low (2.6%) and of limited clinical significance; all affected patients also developed postoperative DVT at different sites; 2) although a considerable proportion of patients developed postoperative DVT, most was distal and asymptomatic; we found one example of symptomatic PE; and, 3) staged bilateral TKA during a single period of hospitalization does not increase the incidence of DVT.

Our findings did not support the hypotheses that a considerable proportion of patients undergoing TKA would have preoperative DVT, or that routine preoperative DVT evaluation would be useful. A previous study found that the preoperative incidence of DVT, diagnosed via Doppler ultrasound, in patients scheduled for TKA was 4.5% [11]. Another study using a 16-row MDCT reported an overall incidence of 8% [17]. As a 128-row MDCT is more accurate than the devices used in earlier studies, we initially hypothesized that the preoperative incidence would be higher than reported previously [23, 24]. However, the incidence of preoperative DVT in our current study was only 2.6%; such DVT was asymptomatic and did not propagate after surgery. In addition, only one patient with preoperative DVT had concomitant varicose veins, which are known to be a risk factor for postoperative VTE [18]. Thus, routine preoperative DVT evaluation did not yield useful information. Furthermore, the preoperative incidence of DVT did not affect the postoperative incidence; the four patients with preoperative DVT exhibited newly developed DVT after surgery. Therefore, preoperative DVT evaluation is of limited clinical utility.

The incidence of DVT in the current study was higher than the incidence previously reported in Asian patients. In a recent meta-analysis, the overall incidence of DVT in Asians was lower than in Westerners [10]. The incidence of postoperative DVT attained 80% in Western patients [25]. In contrast, in a meta-analysis of data on Asian populations, the incidences of overall, proximal, and symptomatic DVT were only 40.4, 5.8, and 1.9%, respectively [10]. A possible explanation for the higher incidence of DVT in the present study is that we used an intramedullary guide during both femoral and tibial resection. Many surgeons use such guides during femoral resection only even if no consensus on the use of guides during tibial resection has yet emerged [26]. Theoretically, an intramedullary guide may be more invasive than an extramedullary guide [27]. However, no study has yet explored whether the incidence of DVT increases after TKA using an intramedullary guide. Furthermore, previous studies on the incidence of DVT do not mention whether intramedullary or extramedullary guides were used during surgery on femora and/or tibiae. Therefore, the issue requires further evaluation.

We found no increase of DVT incidence in the staged bilateral TKA than in the unilateral TKA. In contrast to the hypothesis, unilateral TKA group showed higher DVT incidence per knee. It remains unclear whether simultaneous bilateral TKA is a risk factor for DVT development. When selected patients with DVT symptoms were examined, DVT was more common in those undergoing simultaneous bilateral TKA (12.4% vs. 25.9%) [19]. In contrast, when venography was used to perform routine DVT examinations, no difference in DVT incidence was evident between the unilateral and simultaneous bilateral TKA groups (41.4% of unilateral TKAs vs. 41.8% of simultaneous bilateral TKAs) [20]. In our present study, staged bilateral TKA during a single period of hospitalization does not increase the incidence of DVT. However, patients with DVT in the first-operated limb tended to exhibit DVT in the second-operated limb. This was probably because prior VTE is a risk factor for DVT development [22]. Furthermore, the fact that we found no difference in the incidence of postoperative DVT between the first- and second-operated limbs indirectly suggests that surgery per se is the most significant risk factor for DVT, not prolonged immobilization or hospitalization.

Our study had several limitations. First, our sample size was relatively small and we did not have a control group undergoing pharmacological treatment. However, all patients underwent pre- and post-operative MDCT-indirect venography, which detects DVT more accurately than the methods used in previous studies [18]. Second, we prescribed anticoagulant therapy when DVT was evident on MDCT-indirect venography after surgery. This may have prevented late systematic complications associated with DVT. However, we believe that our findings are still valuable, because we did not prescribe any pharmacological treatment prior to DVT evaluation. In addition, withholding of anticoagulant therapy would have been unethical. Third, since the CT indirect venography was performed on the day 4 after surgery, we were not able to provide the incidence of late onset DVT using this study. Finally, all subjects were Asian, and females predominated. It is well- known that the incidence of overall DVT, those of symptomatic DVT and PE differ between Asians and Westerners. Thus, caution is appropriate when seeking to apply our findings to populations differing in ethnicity and/or sex ratio.

## Conclusion

The incidence of symptomatic DVT was low in Asian TKA patients treated with a mechanical compression device alone, although a considerable proportion of patients exhibited DVT after surgery. Routine preoperative DVT evaluation is probably not required; preoperative DVT was rare and of limited clinical relevance. Furthermore, staged bilateral TKA during a single period of hospitalization did not increase the incidence of DVT.

## Abbreviations

DVT: Deep vein thrombosis
MDCT: Multidetector computed tomography
PACS: Picture archiving and communication system
PCA: Patient controlled analgesia
PE: Pulmonary embolism
ROM: Range of motion
SD: Standard deviation
TKA: Total knee arthroplasty
VTE: Venous thromboembolism
